# PROVIDER PERSPECTIVES ON IMPLEMENTING A PERSON-CENTERED COMMUNICATION TOOL

**DOI:** 10.1093/geroni/igz038.196

**Published:** 2019-11-08

**Authors:** Alexandra Heppner, Nytasia Hicks, Sarina Poth, Kimberly Van Haitsma, Katherine Abbott

**Affiliations:** 1 Miami University, Oxford, Ohio, United States; 2 The Pennsylvania State University, University Park, Pennsylvania, United States; 3 Scripps Gerontology Center at Miami University, Oxford, Ohio, United States

## Abstract

The PAL Card QIP Project sought to understand the intervention characteristics associated with effective implementation. Telephone interviews were conducted with n=26 NH providers who completed the project. Calls were recorded, transcribed verbatim and checked for accuracy. The Consolidated Framework for Implementation Research was utilized as an a priori coding scheme to identify factors associated with effective implementation of the PAL Card project. Major themes emerging from the data related to the evidence strength and quality of the intervention as well as the relative advantage to not assessing preferences (“turns out she doesn't even like TV and we have just been having her watch TV”), adaptability (tailoring for their needs), trialability (expanding offering of intervention after initial success), and complexity of the intervention (sharing the work across departments, difficulty using unfamiliar technology). Providers reported positive intervention characteristics of the PAL cards, however, barriers remain that require additional strategies to successfully implement.

